# 

**Published:** 1888-07

**Authors:** 


The Chicago Medical Journal and Examiner.

Volume 57. Number 1.

### CONTENTS OF THIS NUMBER.

PAGE.

**Original Communications—**

Two Interesting Cases of Skin Disease. E. Wing ..... 1
Binocular Astigmatism. S. O. Richey ..... 4

**Editorials—**

Kolpo-Uretero-Cystotomy ..... 6
The Late Emperor of Germany ..... 10

**Society Reports—**

CHICAGO PATHOLOGICAL SOCIETY—
June Meeting ..... 11
THE GYNÆCOLOGICAL SOCIETY OF CHICAGO—
Regular Meeting, April 20 ..... 13
STATE MEDICAL SOCIETY OF WISCONSIN ..... 36
MICHIGAN STATE MEDICAL SOCIETY ..... 39

**Foreign Medical Societies—**

The Medico-Chirurgical Society of Edinburgh ..... 43

**Extracts and Abstracts** ..... 54-62
**Book Reviews and Notices** ..... 62
**Miscellany** ..... 64
**Announcements** ..... 64
**List of Advertisers** ..... x
**Publisher's Announcements** ..... x

